# Combining Gene–Disease Associations with Single-Cell Gene Expression Data Provides Anatomy-Specific Subnetworks in Age-Related Macular Degeneration

**DOI:** 10.1089/nsm.2020.0005

**Published:** 2020-08-03

**Authors:** Philip J. Luthert, Christina Kiel

**Affiliations:** ^1^ UCL Institute of Ophthalmology, and NIHR Moorfields Biomedical Research Centre, University College London, London, United Kingdom.; ^2^ Systems Biology Ireland and UCD Charles Institute of Dermatology, School of Medicine, University College Dublin, Dublin, Ireland.

**Keywords:** retinal degeneration, gene expression, protein–protein interaction networks, gene ontology

## Abstract

**Background:** Age-related macular degeneration (AMD) is the most common cause of visual impairment in the developed world. Despite some treatment options for late AMD, there is no intervention that blocks early AMD proceeding to the late and blinding forms. This is partly due to the lack of precise drug targets, despite great advances in genetics, epidemiology, and protein–protein interaction (PPI) networks proposed to be driving the disease pathology. A systems approach to narrow down PPI networks to specific protein drug targets would provide new therapeutic options.

**Materials and Methods:** In this study we analyzed single cell RNAseq (RNA sequencing) datasets of 17 cell types present in choroidal, retinal pigment epithelium (RPE), and neural retina (NR) tissues to explore if a more granular analysis incorporating different cell types exposes more specific pathways and relationships. Furthermore, we developed a novel and systematic gene ontology database (SysGO) to explore if a subcellular classification of processes will further enhance the understanding of the pathogenesis of this complex disorder and its comorbidities with other age-related diseases.

**Results:** We found that 57% of the AMD (risk) genes are among the top 25% expressed genes in ∼1 of the 17 choroidal/RPE/NR cell types, and 9% were among the top 1% of expressed genes. Using SysGO, we identified an enrichment of AMD genes in cell membrane and extracellular anatomical locations, and we found both functional enrichments (e.g., cell adhesion) and cell types (e.g., fibroblasts, microglia) not previously associated with AMD pathogenesis. We reconstructed PPI networks among the top expressed AMD genes for all 17 choroidal/RPE/NR cell types, which provides molecular and anatomical definitions of AMD phenotypes that can guide therapeutic approaches to target this complex disease.

**Conclusion:** We provide mechanism-based AMD endophenotypes that can be exploited *in vitro*, using computational models and for drug discovery/repurposing.

## Introduction

Age-related macular degeneration (AMD) is an eye disorder that can cause blurred, distorted, or lost central vision and is the commonest cause of blindness of elderly people.^1^ Patients with early forms of AMD experience reading problems and are additionally very much affected by the uncertainty of not knowing when the onset to late AMD happens—a condition that can occur very suddenly. Late AMD causes major visual impairment that seriously interferes with everyday activities, and affected people describe losing their sight as major distress. Available treatment options are limited (e.g., antivascular endothelial growth factor [VEGF] therapy^2^), of high cost (periodic intravitreal injection) and, notably, they do not provide a cure for AMD.

Significant advances in genetics, epidemiology, and imaging have uncovered some of the molecular mechanisms underlying the pathogenesis of AMD.^3^ The outer retina, including photoreceptor cells, the retinal pigment epithelium (RPE), Bruch's membrane and choroid, including the microvascular supply to the outer retina, the choriocapillaris, all appear to play an important role in the pathogenesis of AMD.

Dysregulation of the complement system appears to be a major factor in many cases of AMD. Based on the latest (and with 16,144 patients the highest sample size) genome-wide association study (GWAS) of AMD,^4^ in addition to nucleotide variants in the major risk gene loci of complement factor H (CFH) (complement pathway) and *ARMS2/HTRA1* (extracellular matrix [ECM] regulation), many small effect variants were identified in genes involved in angiogenesis, lipid metabolism, complement activation, oxidative stress, ECM regulation, and inflammation.

One of the challenges in finding treatments for AMD is that—despite substantial advances in genetics and knowledge about the vast number of pathways and protein–protein interaction (PPI) networks proposed to be involved in the pathogenesis^5^—it is unclear which proteins/pathways and which cells are the optimal drug target(s). Indeed, gene expression data analyses of choroid/RPE and neural retina (NR) tissues obtained from human donor eyes^6^ suggested that most of the genes are not expressed tissue specifically and are indeed expressed in most of the nonretinal body tissues.^5^

In line with this finding, many of the AMD risk gene products (proteins) have proposed roles in generic cell and tissue homeostasis pathways, such as proteostasis, energy homeostasis, mitophagy, autophagy, and choroidal vascular homeostasis,^5^ and are not specifically related to core vision processes (e.g., phototransduction).

Recent technological advances in single-cell RNA sequencing (RNAseq) make it possible to decompose gene expression of tissues into their specific cell types, which provides new avenues for gene-function analyses, especially, new opportunities for systems medicine approaches.

In this study, 2 newly published transcriptome datasets of 10 choroid/RPE^7^ and 7 NR^8^ cell types are used to analyze if some of the AMD risk genes are expressed in specific cells (or subset of cells) only. Furthermore, a genome-wide systematic gene ontology classification database (SysGO) with a one-to-one mapping of genes and predominant gene product function and location is developed, and used to uncover functions and anatomical localizations of AMD risk genes highly expressed in the retina–choroid–complex (RCC). The premise is that risk genes are most likely to exert their effect in cells where they are expressed at high rather than low levels.

This, of course, does not preclude that low expression levels also can have relevance for disease progression. Focusing on highly RCC expressed AMD genes, we reconstruct anatomy-specific PPI subnetworks, which exposes pathways, proteins, and cell types that can be scrutinized for *in vitro* experimental and computational modeling approaches to investigate disease pathogenesis further, and for developing (or repurposing of existing) small molecule and peptide drugs.

## Materials and Methods

### Genome-wide gene ontologies for human protein-coding genes (SysGO)

To refine the association between genes expressed at the back of the eye and relevant cell processes, we chose to develop a novel systematic gene ontology database (SysGO) with a specific emphasis on disease at a tissue as opposed to a solely cellular level. The main aim was to have a functional annotation of genes built and organized from a perspective of function and anatomical localization.

We assume that AMD—and most likely other complex diseases—is a “tissue disease,” where in particular multiple homeostatic mechanisms and the interaction/communication between cell types and extracellular space are perturbed. A list of 19,300 protein-coding gene IDs was obtained from the HGNC (HUGO Gene Nomenclature Committee) database (Supplementary Table S1). As gene name conversions between different published datasets are notoriously difficult, a set of alternative gene names was included. A single gene ontology process/class/function was assigned to each gene product (=protein) based on manual annotations, curated through UniProt information and additional, manual literature interrogation.

As an example to highlight the anatomy-centric gene ontologies, we separately classified transporters (and their subclasses depending on the types of molecules they transport), rather than to include, for example, glucose transporters in carbohydrate metabolism, or amino acid transporters in amino acid metabolism, as the critical property from a systems and tissue perspective is the fact that metabolites are exchanged between cell types.

While it was straightforward to assign a single function per gene for some functions, such as metabolism, organelle functions, cellular machines, cytoskeleton, transporters, adhesion, transcription, and translation, it was more challenging for signaling-related classes. Indeed, signaling proteins tend to be reused and operate in multiple cellular processes. Therefore, to allow for context-specific adaptations, subclasses within the signaling class were often based on the proteins' core catalytic functions (e.g., Ras GTPases, Rho GTPase, guanine nucleotide exchange factors, kinases) or domain compositions (e.g., adaptors, PH-domain containing), rather than their expected roles in cell fates (e.g., proliferation, migration).

SysGO—set 1 was defined by function according to anatomical localization, wherever possible. As such, SysGO classes with prefix_1 (147 classes) are cellular processes including organelles, SysGO classes with prefix_2 (11 classes) are related to ECM organization, SysGO classes with prefix_3 (120 classes) are processes on the tissue level, such as transporters, channels, and receptors, SysGO classes with prefix_4 (14 classes) describe functions related to cell fate changes.

Wherever sensible, SysGO class names were adapted from the Reactome database. In total, 321 classes were defined (“SysGO—set 1”), of which 132 were related to signaling functions.

For easier visualization and for calculating GO class enrichments, some classes were merged (e.g., collagen, serpins elastic fibers, etc.), were grouped into one group (“ECM organization,” actin, tubulin, intermediate filaments, etc.), were grouped into one group (“Cytoskeleton”), resulting in a total of 58 groups (“SysGO—set 2”) (Supplementary Fig. S1). This was further reduced to 15 groups (“SysGO—set 3”) that correspond to the wide-ranged classes of Signaling, Metabolism, Protein translation, folding, modification and degradation, Transcription, Unknown, Cytoskeleton, Organelles, Other, Immune system and Inflammation, Chromatin organization and DNA repair, Neuronal System, synapses, channels, ECM organization, Cell junction and adhesion, Developmental, and DNA Replication.

One thousand three hundred fifty-five genes have an as yet unknown function, of which 1042, however, have a known subcellular localization.

### Subcellular localizations for 19,300 protein-coding genes

A main subcellular localization was assigned for each gene product based on the COMPARTMENTS database,^9^ the Human Protein Atlas,^10,11^ and manual annotations, for example, through UniProt information and additional literature searches. When several subcellular localizations were documented for a protein, the predicted main localization was assigned using the scoring system provided by the COMPARTMENTS database and/or the SysGO annotations.

For example, signaling proteins that could be both in the cytosol and at the plasma membrane but did not have a transmembrane domain (e.g., RAF kinase or scaffolds) were assigned to the cytosol subcellular localization. In total, 47 groups were defined (“SysGO localization—set 1”) (Supplementary Table S1). Merging of some of the groups resulted in 39 (“SysGO localization—set 2”) (Supplementary Fig. S2) or 8 (“SysGO localization—set 3”) different localizations. A predominant subcellular localization was assigned to all but 313 human protein-coding genes.

### Gene expression analysis of 17 cell types in choroid/RPE and NR tissues

Recent human single-cell gene expression datasets of choroid/RPE^7^ and NR^8^ cells were retrieved. The choroid/RPE dataset contained transcript data of Schwann cells (two clusters that were combined in subsequent analyses), melanocytes, endothelial cells, smooth muscle cells, fibroblasts, RPE cells, B cells, T cells/NK cells, monocytes and/or macrophages, and mast cells.^7^ The NR dataset included transcript data of rod photoreceptor cells, cone photoreceptor cells, retinal ganglion cells, horizontal cells, bipolar cells, amacrine cells, and Müller glial cells.^8^ For each of the 17 cell types, the average expression levels across all single cells were calculated.

Gene names were matched to the 19,300 protein-coding genes, and average expression levels for each gene and cell type included in Supplementary Table S1. Next, groups were formed based on the magnitude of gene expression: the top 1% (=193 genes) most highly expressed genes in each of the 17 cell types were identified (“expression group A”). Among the remaining genes, the upper 25% (“expression group B”), the middle 50% (“expression group C”), and the lower 25% (“expression group D”) were identified. Nonexpressed genes were classified as “expression group E,” and genes that could not be mapped or not identified in any of the cell types in the respective two datasets^7,8^ as “expression group F (or N/A)” (3322 genes were not identified in the choroid/RPE dataset and 5838 not in the NR dataset).

To estimate which genes are expressed more specifically to a cell type among all choroid/RPE and NR cell types, respectively, we also calculated a *z*-score for each gene in each tissue, where the gene expression variance across cell types was calculated (*z*-score=[data point − mean]/standard deviation).

### Gene expression analyses in nonretinal tissues

Gene expression classes for nonretinal tissues were obtained from the “Tissue Atlas” resource of the Human Protein Atlas.^10,11^ Information for gene expression classes with regard to abundance and distribution of transcripts across tissues was obtained for 19,300 protein-coding genes, including 9349 genes of class “Detected in all,” 5335 genes of class “Detected in many,” 3176 genes of class “Detected in some,” 668 genes of class “Detected in single,” and 198 genes of class “Not detected” (Supplementary Table S1). Information for 574 genes was not available (N/A).

### Gene expression analysis in choroid/RPE and NR tissue sections

For comparison of gene expression values measured at tissue and single-cell levels, the Whitmore et al. dataset^6^ was used, which contains RNA expression levels from choroid/RPE and NR of four human donor eyes. For each of the tissues (choroid/RPE and NR), the average expression levels across all donors and anatomical locations (nasal, temporal, and macular regions) were calculated. Gene names were matched to the 19,300 protein-coding genes, and average expression levels for each gene and cell type uploaded into Supplementary Table S1.

Similar to the single-cell datasets, groups were formed based on the magnitude of gene expression: the top 1% (=193 genes) expressed genes were identified (“expression group A”). Among the remaining genes, the upper 25% (“expression group B”), the middle 50% (“expression group C”), and the lower 25% (“expression group D”) were identified. Nonexpressed genes were classified as “expression group E” and genes that could not be identified as “expression group F (or N/A).”

### Identification of gene–disease associations

Associations between gene variants and diseases were obtained from the Open Targets Platform, an initiative of the European Bioinformatics Institute. To identify AMD risk genes, we searched for “Age related macular degeneration” and filtered by “Genetic associations” (412 genes; date: January 2020) (Supplementary Table S2). The Open Targets Platform associates a quantitative score to each genes–disease association, where the score depends on factors that affect the relative strength of available evidence, for example, confidence of evidence and sample size for the GWAS data (https://docs.targetvalidation.org/getting-started/scoring). The scoring system ranges from 0 to 1, where the latter represents the strongest association.

Genes associated with other (not classically age related) choroidal and retinal degenerations were obtained by searching for “Retinitis pigmentosa,” “Stargardt disease,” “Congenital stationary night blindness,” “Cone rod dystrophy,” “Leber congenital amaurosis,” “Infantile Refsum disease,” “Usher syndrome,” “Bardet-Biedl syndrome,” “Joubert syndrome,” “Alport syndrome,” and “Cockayne syndrome,” each time filtering for “Genetic associations” (Supplementary Table S3). Genes associated with other age-related diseases were obtained by searching for “Coronary heart disease,” “Diabetes mellitus,” “Rheumatoid arthritis,” “Alzheimer's disease,” “Obesity,” “Multiple sclerosis,” “Asthma,” “Systemic scleroderma,” “Osteoporosis,” and “Parkinson's disease,” and each time filtering for “Genetic associations” (Supplementary Table S4).

### Cell-type-specific AMD protein interaction networks

PPI networks were obtained using the STRING database.^12^ For each highly expressed (groups A and B) risk gene product (=protein), the interacting partners were obtained using settings “Experiments” and “Databases” for the active interaction sources, “Medium confidence of 0.5” for the minimum required interaction score, and “all interactors of the 1st shell” (Supplementary Table S6). Binary PPI from systematic (yeast two-hybrid) screens were retrieved from the HuRi database.^13^ PPI from Affinity-Purification Mass Spectrometry experiments were obtained from the BIOPLEX database.^14^

## Results

### A high-confidence set of genes associated with a risk of developing AMD

**FIG. 1.**
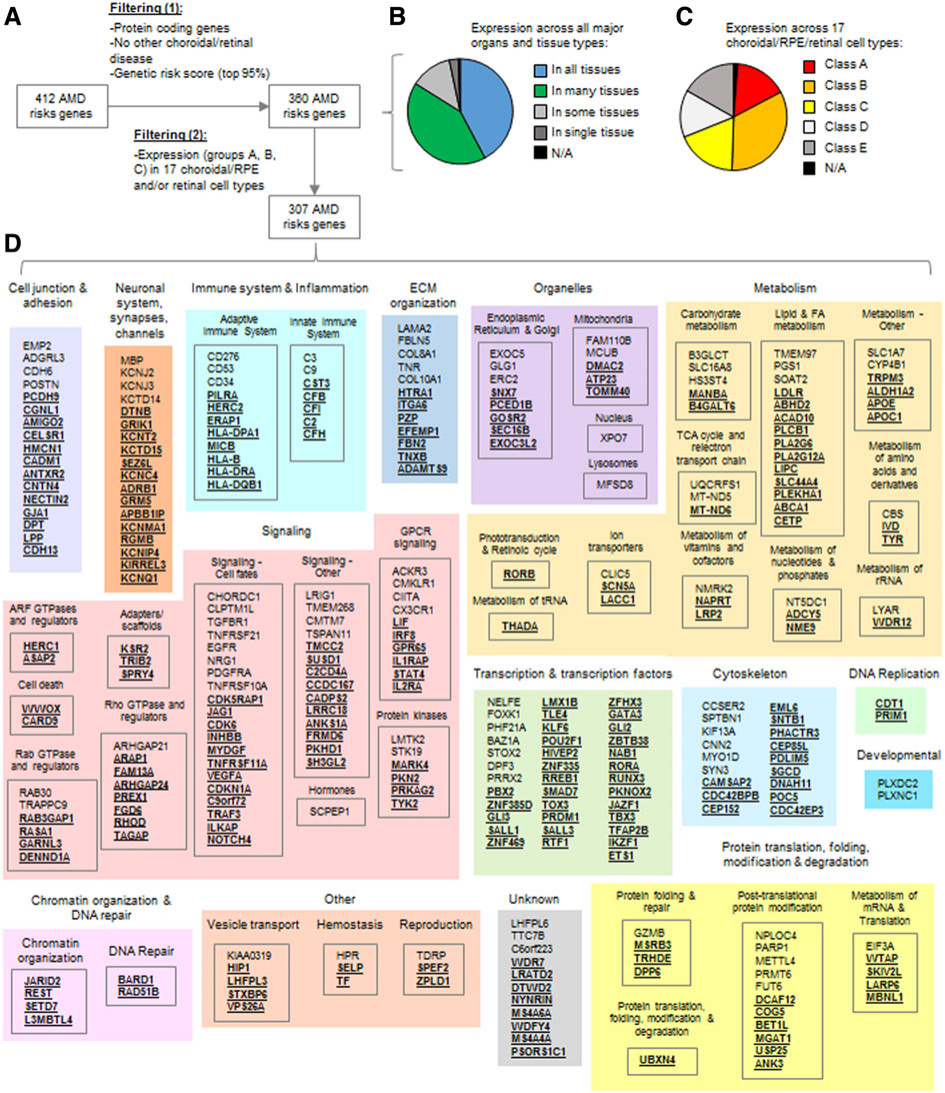
A high-confidence set of genes associated with a risk of developing AMD. **(A)** The workflow for obtaining a high-confidence list of AMD risk genes. **(B)** Gene expression classes for nonretinal tissues of major organ and tissue types of 360 AMD risk genes (obtained from the “Tissue Atlas” resource of the Human Protein Atlas). **(C)** Gene expression classes for 17 choroidal/RPE/retinal cell types of 360 AMD risk genes (A=“expression group A” with the top 1% expressed genes, B=“expression group B” with the upper 25% expressed genes, C=“expression group C” with the middle 50% expressed genes, D=“expression group D” with the lower 25% expressed genes, E=“expression group E” with the nonexpressed genes, and N/A=expression group where genes could not be identified. **(D)** High-confidence list of 307 AMD risk genes sorted by SysGO class. Underlined genes indicate that the genes are involved in at least one other age-related disease. AMD, age-related macular degeneration; RPE, retinal pigment epithelium; SysGO, systematic gene ontology database.

A list of 412 genes linked to AMD based on GWAS studies, and family linkage and candidate gene testing studies of AMD cases versus controls were obtained from the Open Targets Platform (Fig. 1A and Supplementary Table S2). The list of genes was pruned by including only protein-coding genes, removing genes associated with early forms of choroidal and retinal degenerations, and excluding 5% of genes with the lowest risk score (360 genes remained). Gene expression analysis of the reduced set of 360 genes showed that AMD risk genes tend to be expressed in most nonretinal tissues (Fig. 1B), as noticed earlier.^5,15^

Indeed, gene expression analyses of composite choroid/RPE and NR tissues obtained from human donor eyes^6^ showed that AMD genes rarely belonged to “expression group A” (the top 1% of genes with highest transcript abundance), but were more often included in the upper 25% (“group B”), the middle 50% (“group C”), or the lower 25% (“group D”) expression classes (Supplementary Fig. S3).

We reasoned that individual populations of cell types within tissues could express a subset of AMD risk genes at high abundance, which would average out when measuring transcript levels in tissues, but could be detectable in single-cell expression datasets. Therefore, we analyzed recent human single-cell gene expression datasets of choroid/RPE^7^ (containing transcript data of Schwann cells, melanocytes, endothelial cells, smooth muscle cells, fibroblasts, RPE cells, B cells, T cells/NK cells, monocytes and/or macrophages, and mast cells) and NR^8^ (containing transcript data of rod photoreceptor cells, cone photoreceptor cells, retinal ganglion cells, horizontal cells, bipolar cells, amacrine cells, and Müller glial cells). Indeed, a larger fraction of cell types (16%) contained genes among the highest expression group A (Fig. 1C).

The comparison between the above gene expression classes in tissues with single-cell expression data showed a remarkable overall quantitative agreement, where for each tissue expression class a range of expression classes in single cells were observed, which fluctuated around the respective tissue expression class (Supplementary Fig. S3). Summarizing, 58% of the AMD genes were among the top 25% expressed genes (class B) in ∼1 of the 17 choroidal/RPE/NR cell types, and 23 genes were among the top 1% of expressed genes (class A).

The cell types that contained most of the AMD risk genes with expression classes A and/or B were fibroblasts, Schwann cells, endothelial cells, monocytes and/or macrophages, Müller glial cells, and RPE cells, but all cell types studied expressed class A and B genes (Supplementary Fig. S4).

We next filtered the 360 AMD risk genes based on their transcript levels in the 17 choroidal/RPE and/or retinal cell types, and included only those genes that belong to expression classes A, B, or C in ∼1 of the 17 cell types, resulting in 307 genes (Fig. 1D and Supplementary Figs. S5 and S6).

The SysGO classes that were enriched (*p*<0.05 by Fisher's exact test*) over all 19,300 protein-coding genes were the classes of Rho GTPases and regulators (*), cell–cell junction/adhesion (*), protein translation/folding/modification and degradation, neuronal systems/synapses/channels (*), vesicle transport, ECM organization, adaptive immune system, Arf GTPases and regulators, endoplasmic reticulum and Golgi, innate immune system, Ras GTPases and regulators, Signaling—cell fates, Rab GTPases and regulators, protein kinases, and hemostasis (Supplementary Fig. S7A). The SysGO classes are similar to those determined earlier, but our analyses also identified new classes, such as cell–cell junction/adhesion and Rho GTPases and regulators.

An overlap of AMD risk genes with genes associated with other age-related disorders, such as coronary heart disease, diabetes, and Alzheimer's disease, has been noted previously.^5^ Corroborating this, we find a high overlap (70%) of the 307 AMD risk genes with genes of other age-related diseases (Fig. 1D and Supplementary Fig. S8A, B).

The top 5 genes with the highest overlap are the histocompatibility antigen binding proteins HLA-DQB1 and HLA-DRA, IL2RA (regulation of immune tolerance), the transcription factor ETS1 (controlling the expression of cytokine and chemokine genes), and the metalloproteinase ADAMTS9 (in particular controlling endothelial cell–matrix adhesion) (Supplementary Fig. S8C). The functions related to adaptive immunity, inflammation, tissue-general cytokine controlling transcription factors, and endothelial ECM remodeling may explain the systemic effect of some genes and why they could play a role in multiple age-related diseases.

### Retina-choroid-complex specific and widely-expressed AMD risk genes

**FIG. 2.**
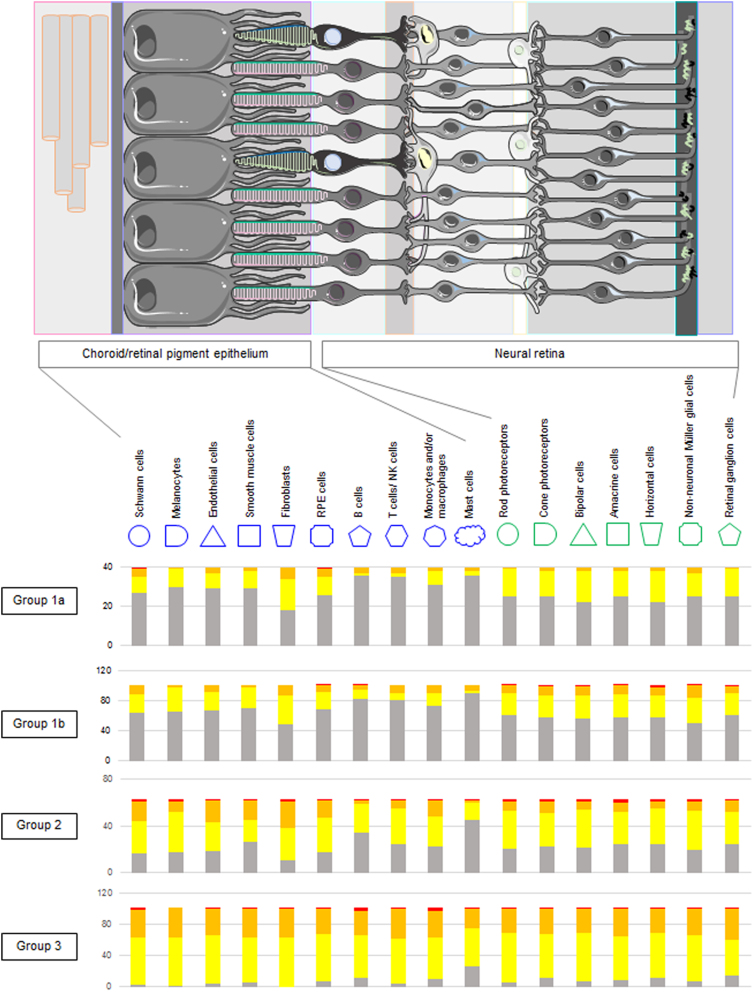
Anatomy of the choroid/RPE and NR tissues and associated cell types considered in this study (upper panel). The figure was prepared using images from Servier Medical Art by Servier, which is licensed under a Creative Commons Attribution 3.0 Unported License. Lower panel: number of genes in groups 1a, 1b, 2, and 3 colored by expression group (red=expression group A, orange=expression group B, yellow=expression group C, and gray=all other expression groups). NR, neural retina.

It is well established that most protein-coding genes are expressed in all tissues and organs.^10^ Indeed, 48% of the 19,300 genes in the SysGO database are expressed in all tissues (Supplementary Table S1). We classified the 307 AMD risk genes into those highly expressed in the RCC (“RCC specific”) as opposed to those expressed in all tissue types (“tissue generic”) (Supplementary Table S5). The RCC-specific groups were created by first removing all genes not belonging to the tissue expression class “Detected in all,” and by further distinguishing genes that were not found in other age-related diseases (=group 1a; 40 genes) and those that are also implicated in other age-related diseases (=group 1b; 102 genes) (Fig. 2).

Further, two groups, one with intermediate tissue specificity (with or without implication in other age-related diseases) (=group 2; 63 genes) and one with generalized tissue expression (with or without implication in other age-related diseases) (=group 3; 102 genes) (Fig. 2) were defined. After mapping gene expression classes obtained from the analysis of single-cell RNAseq onto the four different groups of AMD genes, we found that groups 1a and 1b had the smallest fraction of genes in the highest expression classes (A and B), and those fractions slightly increased for groups 2 and 3. However, as expected for the generic genes, the fraction of genes that increased most for groups 2 and 3 belonged to expression class C (medium 50% expressed genes) (Fig. 2).

Next, SysGO was used to characterize functional enrichments among the cell specificity groups. The SysGO classes (set 2) that were enriched (*p*<0.05 by Fisher's exact test*) over all 19,300 protein-coding genes for group 1a were cell–cell junction/adhesion (*), ECM organization (*), hemostasis, carbohydrate metabolism, metabolism of vitamins and cofactors, ion transporters (among others) (Supplementary Fig. S5B).

Group 1b was enriched in the classes of neuronal systems/synapses/channels (*), cell–cell junction/adhesion (*), Rho GTPases and regulators, phototransduction and retinoic cycle, Arf GTPases and regulators (among others) (Supplementary Fig. S7C).

Group 2 was enriched in the classes of Rho GTPases and regulators, ECM organization, metabolism of rRNA, adaptive immune system, cell–cell junction/adhesion (among others) (Supplementary Fig. S7D).

Group 3 was enriched in the classes of protein translation/folding/modification and degradation (*), protein kinases, Rho GTPases and regulators, Rab GTPases and regulators, lysosomes, and Arf GTPases and regulators (among others) (Supplementary Fig. S7E).

Overall, when considering the SysGO set 3 classes, we found the trend that the cell-type-specific groups 1a and 1b tend to be associated with tissue and cell–tissue communication functions, such as cell–cell adhesion, ECM organization, immune system, metabolism, channels, transporters, and membrane receptors, while the cell/tissue-general groups 2 and 3 tend to be associated with core cell signaling functions, small GTPases, protein kinases, protein translation, folding, modification and degradation, and chromatin organization. Thus, the RCC-specific AMD endophenotype is likely one that manifests as a consequence of perturbations of cell communication, tissue integrity, and metabolism.

### RCC-specific AMD risk genes and subcellular localization

**FIG. 3.**
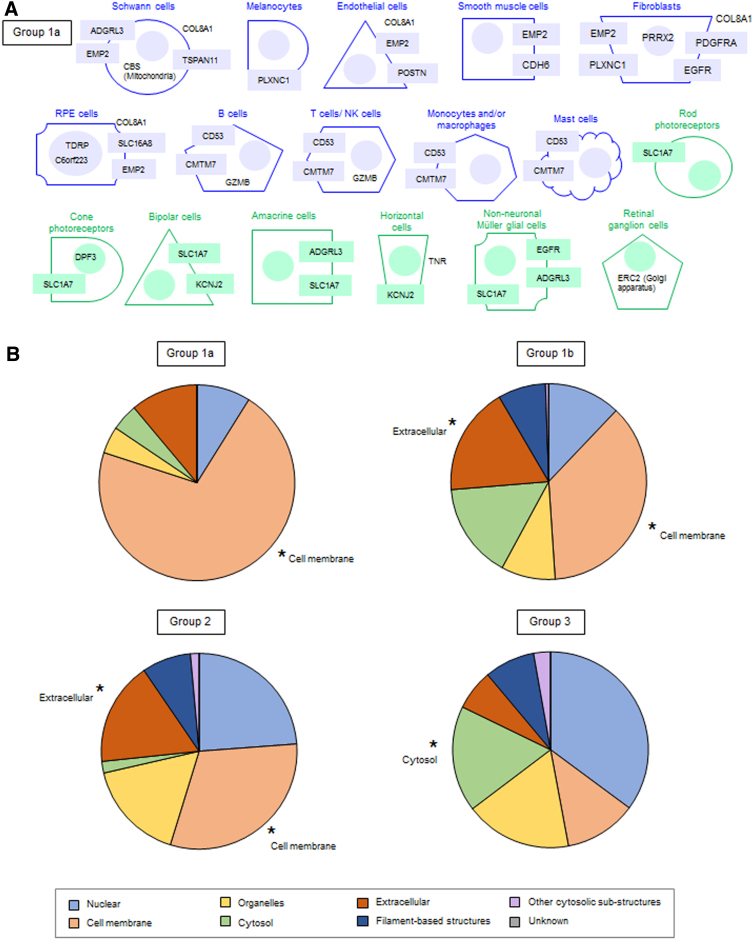
Subcellular localization of 307 high-confidence AMD risk genes. **(A)** Subcellular localization of group 1a genes that are highly expressed (expression groups A and B) in 17 cell types. Each cell type is represented using a distinct geometrical shape and color (blue=choroid/RPE cells and green=NR cell types) (Fig. 2). Filled squares indicate membrane association, gene names outside the cell indicate extracellular localization, those inside the cell indicate cytosolic localization, and those within the filled circle indicate nuclear localization. Other cellular/organelle localizations are indicated. **(B)** Subcellular localizations for all genes in groups 1a, 1b, 2, and 3. The color code corresponds to the subcellular localizations used in Supplementary Figure S2.

To obtain further insights into the molecular functions of cell-type-specific AMD risk genes, we focused on the highly expressed gene groups A (top 1% expressed) and B (upper 25% expressed). In agreement with the SysGO functional classes described above (e.g., cell adhesion, transmembrane proteins, channels, transporters), group 1a was highly enriched in genes localized at the plasma membrane (PM)—in particular at the PM of fibroblasts, Schwann cells, and Müller glial cells (Fig. 3A, B).

This reinforces the hypothesis that cell-type-specific AMD risk genes are associated with a dysregulation of cell–cell communication. Group 1b was also highly enriched for membrane as main subcellular localization, but additionally genes were enriched for an extracellular localization (Fig. 3B and Supplementary Fig. S9). The extracellular space has the capacity to communicate between tissues; hence, this may point to systemic factors and the overlap of genes with other age-related diseases.

In contrast, genes of group 2 were only slightly enriched for PM and extracellular localization (Fig. 3B), and group 3 has a very similar subcellular distribution (Fig. 3B) as all 19,300 protein-coding genes (Supplementary Fig. S2). Altogether, we propose that understanding/targeting proteins of group 1a is the way forward for the definition of AMD-specific endophenotypes, while genes/proteins of group 1b should be studied for targeting/promoting “healthy aging.”

### Anatomy-specific PPI networks of AMD

To further investigate the underlying mechanisms of AMD disease pathogenesis, and to characterize the anatomical localization and functioning of AMD risk genes of group 1a, we reconstructed PPI networks for the 22 highly expressed genes (expression groups A and B) in ∼1 of the 17 cell types (see Materials and Methods section; Supplementary Table S6).

To focus on interaction partners that are not widely expressed in different cells and tissues, the interactors were filtered to include only those that are (i) highly expressed in at least one cell type (expression groups A and B), and (ii) not belonging to class “Detected in all tissues” based on the Human Protein Atlas (342 interactors). Based on the SysGO classes, subcellular localization, further manual literature searches, and/or SysGO classes of interactors, we mapped the 22 risk proteins and their PPI onto 6 anatomical layers (ALs) (note that they are spatially organized rather like the layers of an onion).

This included a further removal of interactors that were in disconnected anatomical compartments that would prevent proteins from interacting (e.g., extracellular and cytosol). We also highlighted genes that have a high *z*-score in a particular cell type (compared with the other cell types), as this information about a more cell-type-specific expression could further guide follow-up experiments and potential therapeutic approaches.

#### AL1: immune and other systemic influences

**FIG. 4.**
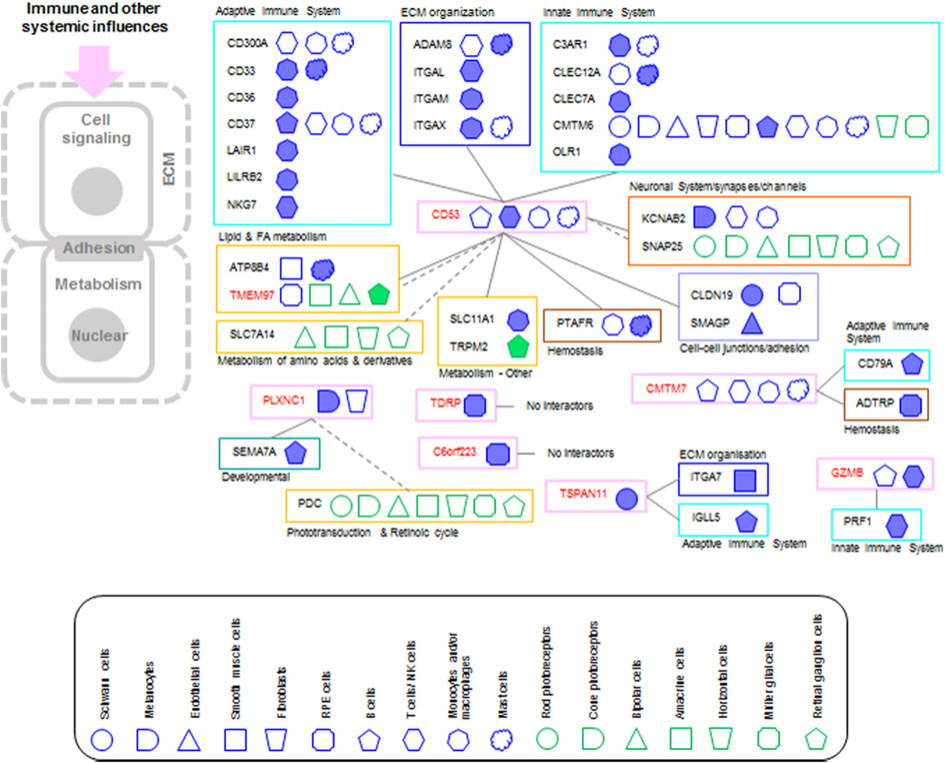
PPI networks of AMD risk protein of the AL1. AMD risk proteins are indicated in red and the color around the name corresponds to the AL. Interactors are grouped based on SysGO classes and linked to the AMD risk protein. The cell types in which the AND risk genes are highly expressed (expression groups A and B) are indicated using the geometrical shapes used before and as indicated in the legend. Filled geometrical shapes indicate *z*-scores of ≥2 for a specific gene when compared with its gene expression in the other cell types of the respective dataset (Voigt or Liang). AL, anatomical layers; PPI, protein–protein interaction.

Seven AMD risk proteins and 33 interactors were included in this AL (Fig. 4). CD53, an adaptive immune system protein expressed in B cells, T cells/NK cells, monocytes and/or macrophages, and mast cells, has the highest number of interaction partners. Many of those interactors are also expressed in the CD53-expressing cell types, and their SysGO classes, in addition to the adaptive immune system, are innate immune system, ECM organization, cell–cell junction/adhesion, and metabolism. CMTM7 has cytokine activity and is expressed in B cells, T cells/NK cells, monocytes and/or macrophages, and mast cells.

The SysGO classes of the two interactors are adaptive immunity (CD79A, expressed in B cells) and hemostasis (ADTRP, expressed in RPE cells). GZMB functions in the cell lysis of target cell in immune responses, and is expressed in B cells and T cells/NK cells. Its interactor, PRF1, is also expressed in T cells/NK cells and has a role in the innate immune system. Granzyme inhibitors are expressed at barrier sites and endothelium, potentially to prevent granzyme-mediated damage, which may indicate a trade-off between immune activation and cellular damage.^16^

TSPAN11, a protein with unknown function, was included in AL1, as the two interacting proteins are involved in adaptive immunity (IGLL5, expressed in B cells) and ECM organization (ITGA7, expressed in smooth muscle cells).

C6orf223, a protein with unknown function, is expressed in RPE cells, and no PPI were identified. Interestingly, however, single nucleotide polymorphisms in this gene are linked to a variation in circulating VEGF levels,^17^ thus could explain the contribution to the AMD phenotype. Testis development-related protein, only known to contribute to sperm motility (Uniprot), is highly expressed in RPE cells, but no PPI were identified. No eye/retina-related functions are described for this protein, except a GWAS association with “eye inflammation” (Open Targets platform), which was the reason this protein was assigned to AL1. PLXNC1 is a receptor for SEMA7A and expressed in melanocytes, and fibroblasts. It appears to be proinflammatory acutely,^18^ but enhances fibrosis,^19^ thus may explain its role in AMD.

#### AL2: tissue/ECM organization

**FIG. 5.**
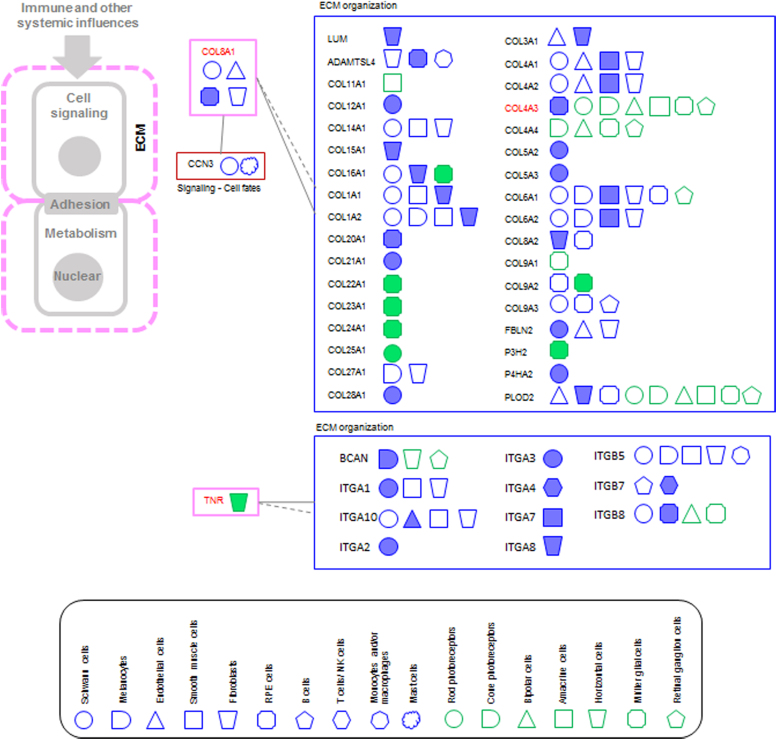
PPI networks of AMD risk protein of the AL2. AMD risk proteins are indicated in red and the color around the name corresponds to the AL. Interactors are grouped based on SysGO classes and linked to the AMD risk protein. The cell types in which the AND risk genes are highly expressed (expression groups A and B) are indicated using the geometrical shapes used before and as indicated in the legend. Filled geometrical shapes indicate *z*-scores of ≥2 for a specific gene when compared with its gene expression in the other cell types of the respective dataset (Voigt or Liang).

Two AMD risk proteins and 46 interactors were included in this group (Fig. 5). COL8A1 is an ECM component that is needed for migration and proliferation of vascular smooth muscle cells, and therefore likely involved in vessel wall integrity (UniProt). It is expressed in Schwann cells, endothelial cells, fibroblasts, and RPE cells. As expected, most of its interactors belong to SysGO class ECM organization (collagens) and are also expressed in choroid/RPE cell types.

However, many collagens are expressed also in NR cell types, and it is unclear if the noncross-linked secreted procollagen molecules expressed in choroid/RPE cells can migrate to distant regions in the NR, heterodimerize and crosslink there. There is an intriguing link to changes in expression of COL8A1 following skin remodeling after microneedling, which also leads to changes in expression of tissue inhibitor of metalloproteinases (TIMP)3, a gene in which mutations are causal for Sorsby's fundus dystrophy, a phenocopy of AMD.^20^ Possibly, there is a ECM–protein/matrix metalloproteinase/TIMP network. COL8A1 is also one of the “sprouting angiogenesis” genes with delayed expression after stroke in aged rats,^21^ providing a link to choroidal angiogenesis.

Tenascin-R (TNR) is a neural ECM component and expressed in horizontal cells, which raises the possibility that horizontal cells interact with a population of microglia^22^ in the outer plexiform layer. All interactors of TNR belong to SysGO class ECM organization; however, several of the proteins are highly expressed in non-neuronal cell types.

#### AL3: tissue/cell–cell adhesion

**FIG. 6.**
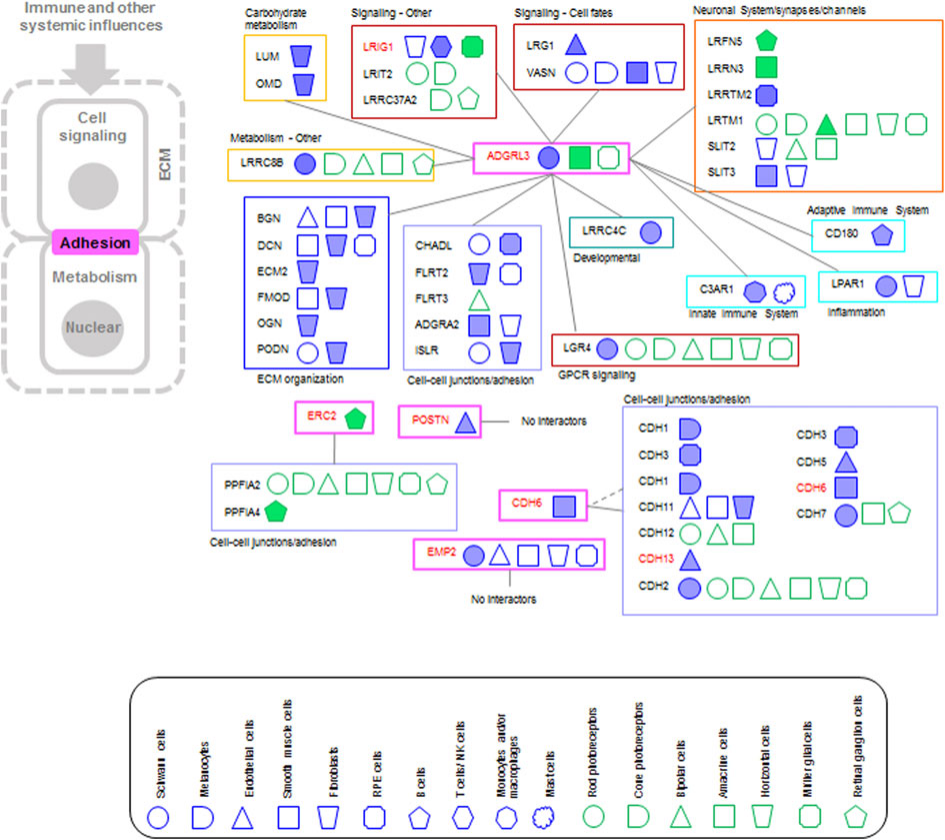
PPI networks of AMD risk protein of the AL3. AMD risk proteins are indicated in red and the color around the name corresponds to the AL. Interactors are grouped based on SysGO classes and linked to the AMD risk protein. The cell types in which the AND risk genes are highly expressed (expression groups A and B) are indicated using the geometrical shapes used before and as indicated in the legend. Filled geometrical shapes indicate *z*-scores of ≥2 for a specific gene when compared with its gene expression in the other cell types of the respective dataset (Voigt or Liang).

Four AMD risk proteins and 43 interactors were included in this group (Fig. 6). Most of the interactors bind to ADGRL3, a protein involved in cell–cell adhesion and neuron guidance, which is highly expressed in Schwann cells, amacrine cells, and microglia. Indeed, most interactors of ADGRL3 belong to the SysGO classes of cell–cell junction/adhesion and neuronal system/synapses/channels, but also of ECM organization, signaling, metabolism, and immune system and inflammation.

CDH6 belongs to the cadherin family, which are calcium-dependent cell adhesion proteins, and is highly expressed in smooth muscle cells. CDH6 has a role in NOTCH3 signaling with links to a vasculopathy arising from changes in pathways related to HTRA1.^23^ Therefore, this protein potentially has important interactions with the HTRA1-associated pathology in AMD. EMP2 plays a role in cell migration and adhesion (of endothelial cells), and is highly expressed in Schwann cells, endothelial cells, smooth muscle, fibroblasts, and RPE.

ERC2 is proposed to be involved in the organization of the cytomatrix at the nerve terminals (Uniprot) and is expressed in retinal ganglion cells. Indeed, ERC2s interactors PPFIA2 and PPFIA4 function in cell–cell adhesion and are expressed in several NR cell types. POSTN, highly expressed in endothelial cells, has no known interactors, but has been shown to promote fibrovascular scar formation.^24,25^

#### AL4: tissue/cell signaling

Four AMD risk proteins localized at the plasma membrane and 57 extracellular interactors were included in this AL (Supplementary Fig. 10). Epidermal growth factor receptor (EGFR) and platelet-derived growth factor receptor alpha (PDGFRA) are transmembrane receptors that are highly expressed only in one cell type (fibroblasts of the choroid). We included all extracellular interactors for EGFR and PDGFRA expressed in choroid/RPE cell types, but not those that were exclusively expressed in NR cell types, assuming that paracrine signaling factors that bind those receptors on fibroblasts are confined within the choroid/RPE tissue layer.

EGFR-mediated signaling in fibroblasts is important for repair responses, and aging fibroblasts (in skin) were shown to lose their epidermal growth factor responsiveness due to decreased EGFR levels, which resulted in impaired wound healing.^26^

Likewise, PDGFRA plays a role in cell migration and wound healing, and the link to AMD might be through formation of disciform scars. KCNJ2 is a potassium channel expressed in bipolar and horizontal cells, but so far there is no evidence that links these cells to AMD.

SLC1A7 is a transporter for l-glutamate, and expressed in rod and cone photoreceptors, bipolar cells, amacrine cells, and microglia. Its association with chloride conductance may reflect a role in visual transduction.

#### AL5: tissue/metabolism

Two AMD risk proteins, CBS and SLC16A8, were included in this AL (Supplementary Fig. 11A). CBS is an enzyme catalyzing the first step of the trans-sulfuration pathway to eliminate the toxic metabolite l-homocysteine, and is highly expressed in Schwann cells. Indeed, this enzyme is associated with several eye disorders.^27^ SLC16A8 is a plasma membrane transporter that catalyzes the transport of monocarboxylates such as lactate and pyruvate, and is highly and specifically expressed in RPE cells.

Recently, a “metabolic ecosystem” between RPE and photoreceptor cells was proposed, where lactate is a key metabolite.^28^ According to this model, glucose from the choroid is passed through the RPE to the photoreceptor, where it is metabolized to lactate, which is in turn taken up by the RPE and used as a fuel molecule. Thus, risk variants associated with the SLC16A8 lactate transporter may dysregulate this metabolic ecosystem, thereby contributing to the AMD phenotype.

#### AL6: cell/signaling and nuclear

This AL, like AL4, contains the receptors EGFR and PDGFRA expressed in fibroblasts, but the interactors are pruned for intercellular proteins (expressed in fibroblasts) (Supplementary Fig. 11B). The SysGO terms of the 11 interactors belong to signaling-related functions, but also cytoskeleton, and protein folding and repair functions. AL6 also contains again KCNJ2, the potassium channel expressed in bipolar and horizontal cells, but here focusing on intracellular interactors, which link to G protein signaling and other synapse- and channel-related functions.

With respect to nuclear proteins, DPF3 belongs to the neuron-specific chromatin remodeling complex and is expressed in cone photoreceptor cells. It is unclear how this protein may contribute to the AMD phenotype. PRRX2 is highly expressed in choroidal fibroblasts, and an upregulation of PRRX2 in (cardiac mice) fibroblasts was described after myocardial infarction.^29^

Altogether, our integrated analysis of PPI and SysGO provides an anatomic description of AMD disease pathology, which is clearly dominated by proteins in cell membrane and extracellular compartment, qualifying AMD as disease of perturbation of tissue homeostasis and communication between cell types. It also enables follow-up studies that aim to quantitatively characterize the molecular mechanism in more detail, for example, using computational whole cell model approaches.^30^

Furthermore, it can guide therapeutic options by taking subcellular localizations into consideration, which can define the therapeutic approach. Furthermore, information about cell-type-specific *z*-scores can inform the therapeutic approach: a gene expressed in one or few cell types might be better targeted with a cell-specific approach (gene therapy), while a gene that is expressed in multiple cells might be better targeted with choroidal drug delivery systems.

## Discussion and Conclusions

The primary aim of this study was to combine the richness of genetic risk data for AMD with recently published single-cell RNAseq data to highlight insights into the pathogenesis of this disorder. While much is known about the disease, and good interventions exist for the vascular complications, we are still unable to prevent early disease, largely asymptomatic progressing to late blinding disease. The majority of the genetic risk for AMD is known to be associated with gene related to the alternate complement pathway (notably CFH) and the ARMS2/HTRA1 locus. These certainly make logical targets for therapy, although to date trials of complement inhibitors have been disappointing and the way in which polymorphisms at ARMS2/HTRA1 influence disease remains unknown, confounding attempts to develop a treatment.

We have elected to take a more system-wide approach and embraced the wide range of genes associated with AMD risk as defined by the “Open Targets” procedural method. In this way, we sought to gain an appreciation of the disease as a whole. We chose to integrate this with single-cell gene expression data because, although the RPE is widely regarded as the focus of disease in AMD, other cell types in the choroid have been implicated (particularly the choriocapillaris endothelium), and the proximity of different cell populations in the choroid offers the potential for important cell–cell interactions that may govern disease progression.

The importance of a systems approach to AMD has been emphasized in a recent article by Handa et al.^31^ and builds on other important investigations of gene expression in AMD.^32^

To facilitate the context-specific interpretation of the patterns of gene expression, we developed a novel gene expression ontology, SysGO, which can be used independently or alongside the well-established gene ontologies.^33^ While there cannot be a definitive classification of genes, SysGO has been highly effective in our hands, as it enables rapid filtering of genes with specific functions and subcellular localizations, and includes the most up-to-date gene names for 19,300 protein-coding genes.

SysGO was developed with a tissue description of disease pathology in mind, highly relevant for AMD pathogenesis, and this will likely also hold for other age-related complex diseases. As such, SysGO might be useful in other disease contexts, including cancer.

An unexpected finding came from the exploration of disease-specific genes and those also associated with other age-related disorders. There is a strong emphasis on cell–cell adhesion and communication with, as the group of genes associated with many age-related disorders are included, an emphasis on ECM. Others have argued that, for instance, elastin degradation is a common feature of several age-related phenomena,^34^ and we have shown that it is a feature of the AMD pathology in the more localized context of Bruch's membrane.^35^ On the basis of the findings in this study, we propose that a greater emphasis should be given to the identification of cell-surface and ECM targets.

The predominance of extracellular space and cell-surface gene products linked to AMD pathogenesis raises the important question as to how these observations fit with the critical genetic risk determined by specific haplotypes in *CFH*. The speculation here is that there may be specific interactions between a generic failure of CFH to bind to and protect host and extracellular and cell-surface targets of attack by the innate and probably also adaptive immune systems as we age.

The way in which we utilized the Open Targets data did not discriminate between AMD progressing to geographic atrophy, which is increasingly recognized as being the natural history end-point of the disease, and choroidal neovascularization, which in many individuals appears to intervene in a stochastic way leading to earlier often sudden impairment of vision. Some of the genes identified, for instance C6orf223, nevertheless would appear to be more likely related to neovascular disease.

Beyond AMD, our findings raise issues in relation to aging and other age-related disorders that warrant further investigation. The grouping of genes that are broadly associated with other age-related conditions might offer exciting opportunities for therapeutic targets that impact the progression of a wide range of aging disorders. It would also be interesting to learn to what extent other age-related conditions are dominated by genes linked to cell–cell interactions (adhesion and other forms of communication). The, although speculative, hypothesis might be that age-related disorders can be in part considered the combination of a set of generic aging genetic risks that interact with tissue/cell-specific cell-surface risks.

When considering cellular targets for AMD, the emphasis has, quite reasonably, been on the RPE. The RPE sits in an environment prone to oxidative stress, it sustains a high metabolic burden supporting the photoreceptors and, for instance, is known to accumulate mitochondrial damage.^36^ There is also considerable interest in the possibility that the choriocapillaris endothelium is a primary target for complement-mediated damage.^7^ This study, however, highlights the possibility that other cells within the choroid play a significant role.

We know relatively little of the interactions between choroidal melanocytes, fibroblasts, and the choroidal circulation as a whole and the RPE. Ultimately, a cell systems description of the choroid will require a much deeper understanding of these interactions as well as a framework for their description and for defining their potential role(s) in disease. As discussed above, important elements of such a framework will include descriptions of the ECM and the complexity of immune/inflammatory processes. A further integrating thread is metabolism.

The metabolic interplay between RPE and photoreceptors is becoming increasingly well characterized with what would have previously been a surprising level of integration between the two classes of cells.^28^ This “ecosystem” needs to be integrated into the broader environment of the choroid, its circulation, and the various systemic factors that influence the choroid and retina for us to have a comprehensive understanding of AMD.

In conclusion, the major genetic risks for AMD involving the complement pathway and the *HTRA1/ARMS2* locus offer exciting possibilities for therapy. But as with other age-related degenerative disorders, AMD is a complex condition that may require a systems approach to identify the key therapeutic target(s). Combining rich datasets of genetic risk with single-cell studies of gene expression, together with PPI networks, offers an exciting approach to develop systems understanding of disease.

## Supplementary Material

Supplemental data

Supplemental data

Supplemental data

Supplemental data

Supplemental data

Supplemental data

Supplemental data

Supplemental data

Supplemental data

Supplemental data

Supplemental data

Supplemental data

Supplemental data

Supplemental data

Supplemental data

Supplemental data

Supplemental data

## Abbreviations Used

AL: anatomical layers
AMD: age-related macular degeneration
CFH: complement factor H
ECM: extracellular matrix
EGFR: epidermal growth factor receptor
GWAS: genome-wide association study
HGNC: HUGO Gene Nomenclature Committee
N/A: not available
NR: neural retina
PDGFRA: platelet-derived growth factor receptor alpha
PM: plasma membrane
PPI: protein–protein interaction
RCC: retina–choroid–complex
RNAseq: RNA sequencing
RPE: retinal pigment epithelium
SysGO: systematic gene ontology database
TIMP: tissue inhibitor of metalloproteinases
TNR: Tenascin-R
VEGF: vascular endothelial growth factor
