# Supplementary material for: Combining Gene–Disease Associations with Single-Cell Gene Expression Data Provides Anatomy-Specific Subnetworks in Age-Related Macular Degeneration
Source: Netw Syst Med. 2020 Aug 3;3(1):105–21. doi: 10.1089/nsm.2020.0005 (PMC7416628; doi:10.1089/nsm.2020.0005)
Supplement: Supplemental data [file Supp_Fig1.pdf]

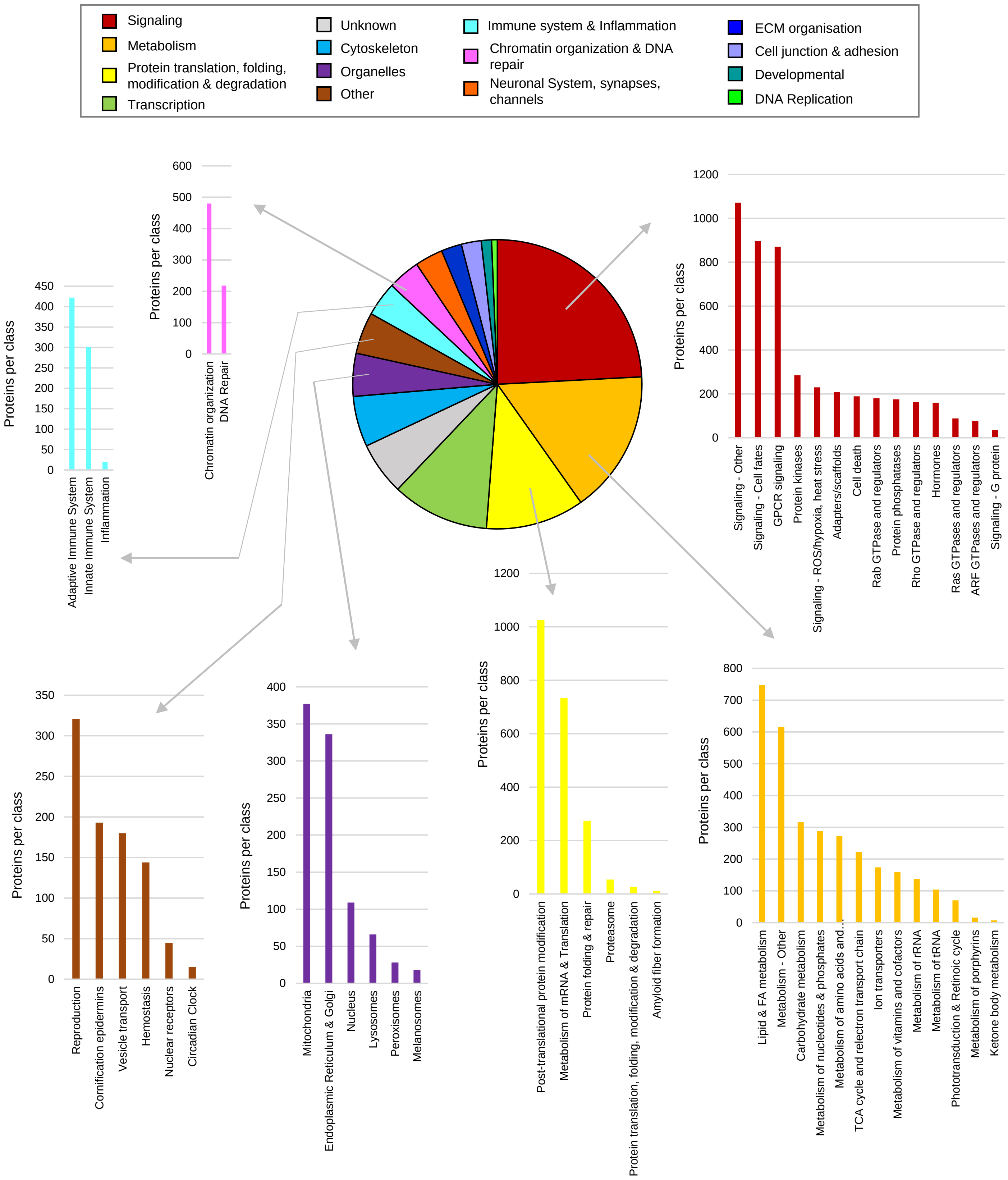

Supplementary Fig. S1. Functional classes statistic for 19300 protein-coding genes of the SysGO database. In total, 321 classes were defined (“SysGO – set 1”) (Supplementary Table S1). For easier visualisation, some SysGO classes were merged, resulting in a total of 58 groups (“SysGO – set 2”), which are shown here. For colouring purposes, those functional classes were grouped into 15 groups (“SysGO – set 3”).
