# Supplementary material for: Combining Gene–Disease Associations with Single-Cell Gene Expression Data Provides Anatomy-Specific Subnetworks in Age-Related Macular Degeneration
Source: Netw Syst Med. 2020 Aug 3;3(1):105–21. doi: 10.1089/nsm.2020.0005 (PMC7416628; doi:10.1089/nsm.2020.0005)
Supplement: Supplemental data [file Supp_Fig2.pdf]

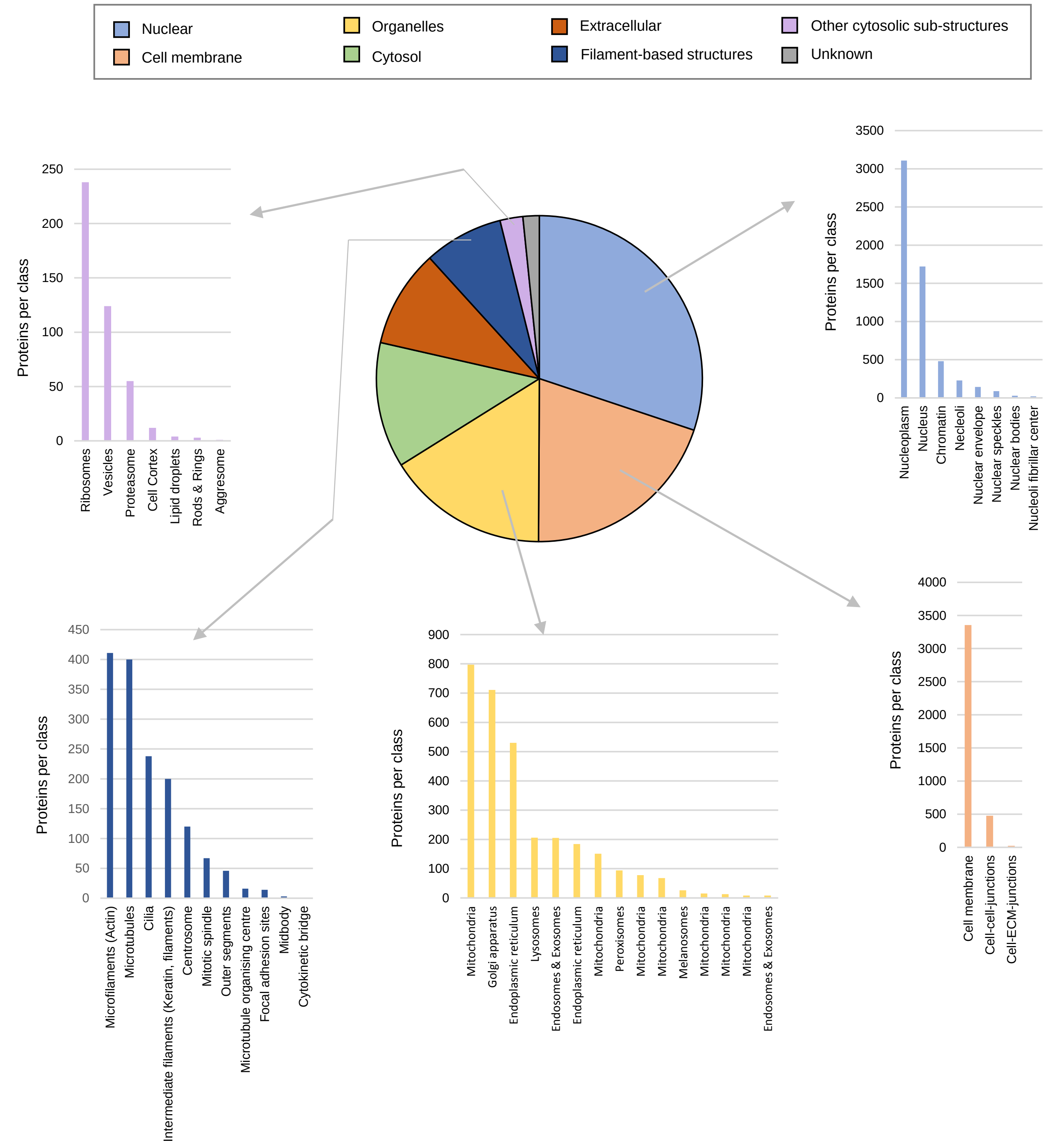

Supplementary Fig. S2. Subcellular localisation statistic for 19300 protein-coding genes of the SysGO database. In total, 47 groups were defined ("SysGO localisation – set 1") (Supplementary Table S1). Merging of some of the classes resulted in 39 groups ("SysGO localisation – set 2"), which are represented here and coloured according to 8 super-classes ("SysGO localisation – set 3").
