# Supplementary material for: Combining Gene–Disease Associations with Single-Cell Gene Expression Data Provides Anatomy-Specific Subnetworks in Age-Related Macular Degeneration
Source: Netw Syst Med. 2020 Aug 3;3(1):105–21. doi: 10.1089/nsm.2020.0005 (PMC7416628; doi:10.1089/nsm.2020.0005)
Supplement: Supplemental data [file Supp_Fig3.pdf]

A

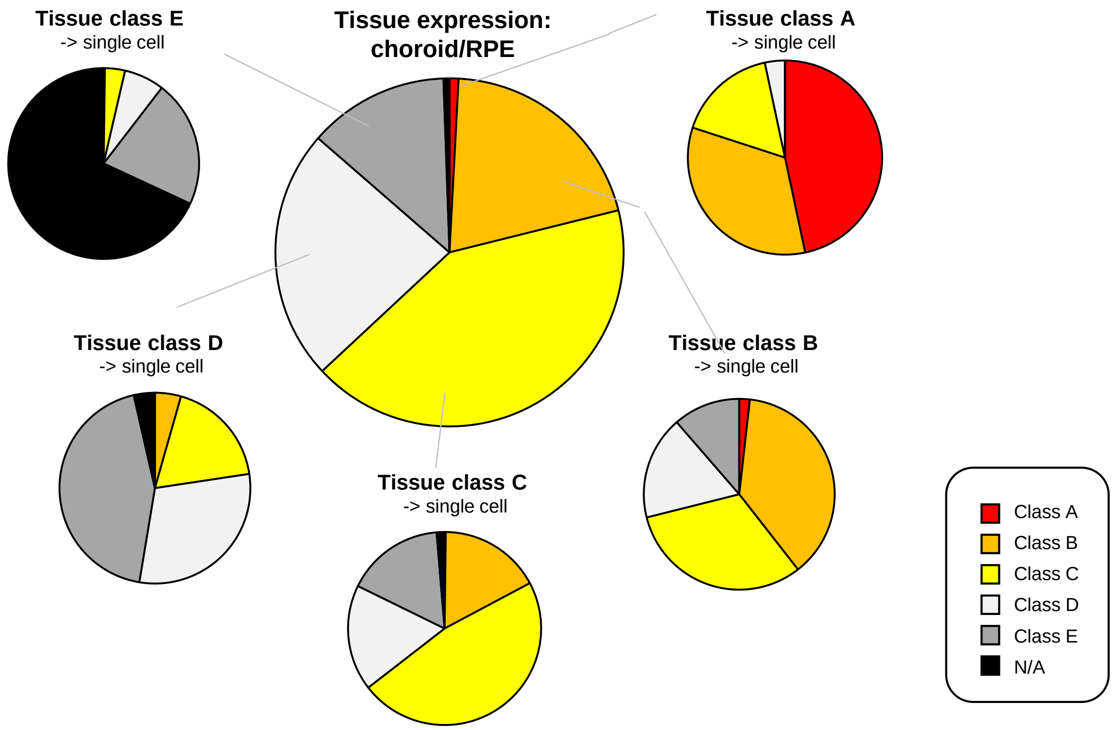

B

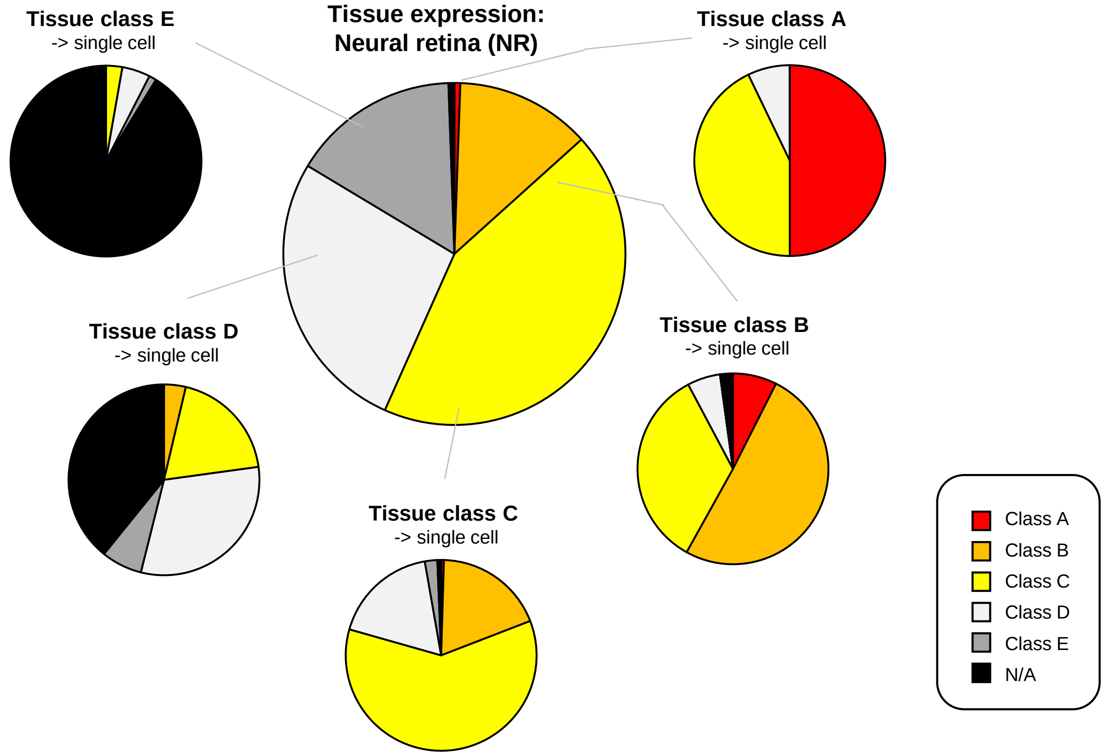

Supplementary Fig. S3. Comparing gene expression classes of tissues with those of single cells. Comparing expression classes in choroid/RPE (**A**) and neural retina (**B**) (Whitmore et al, 2014) with expression classes in single cells (Voigt et al, 2019 and Liang et al, 2019). The top 1% (=193 genes) expressed genes belong to “expression group A”, the upper 25% to “expression group B”, the medium 50% to “expression group C”, and the lower 25% to “expression group D”. Not expressed genes were classified as “expression group E” and genes that could not be identified were grouped into “expression group F (or N/A)”.
