# Supplementary material for: Combining Gene–Disease Associations with Single-Cell Gene Expression Data Provides Anatomy-Specific Subnetworks in Age-Related Macular Degeneration
Source: Netw Syst Med. 2020 Aug 3;3(1):105–21. doi: 10.1089/nsm.2020.0005 (PMC7416628; doi:10.1089/nsm.2020.0005)
Supplement: Supplemental data [file Supp_Fig4.pdf]

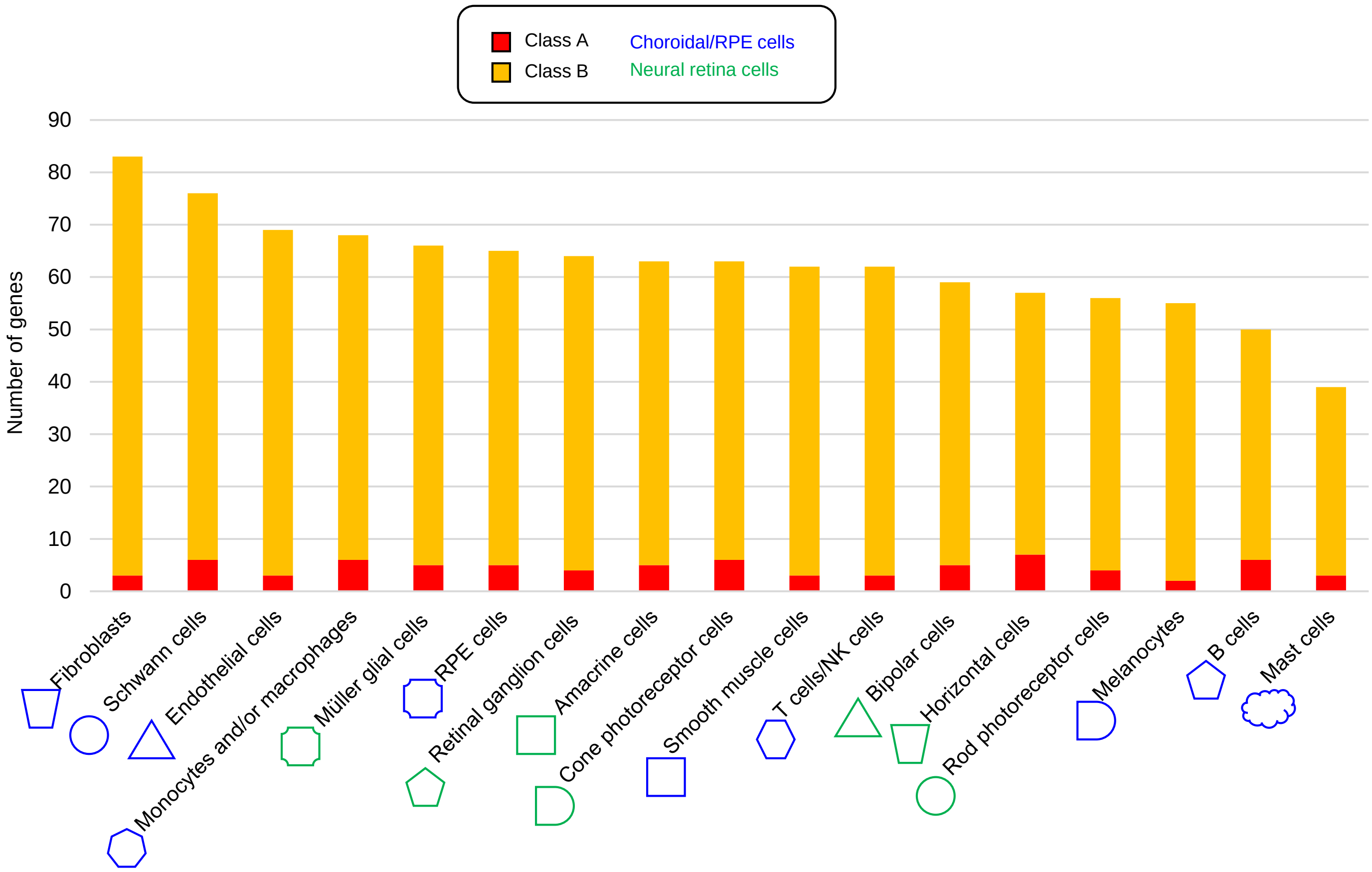

Supplementary Fig. S4. The choroidal/RPE and neural retina cell types that contain most of the highly expressed AMD risk genes. The number of AMD genes with expression classes A (top 1% expressed genes) or B (upper 25 % expressed genes) are shown based on single cell RNAseq data (Voigt et al, 2019 and Liang et al, 2019).
