# Supplementary material for: Combining Gene–Disease Associations with Single-Cell Gene Expression Data Provides Anatomy-Specific Subnetworks in Age-Related Macular Degeneration
Source: Netw Syst Med. 2020 Aug 3;3(1):105–21. doi: 10.1089/nsm.2020.0005 (PMC7416628; doi:10.1089/nsm.2020.0005)
Supplement: Supplemental data [file Supp_Fig5.pdf]

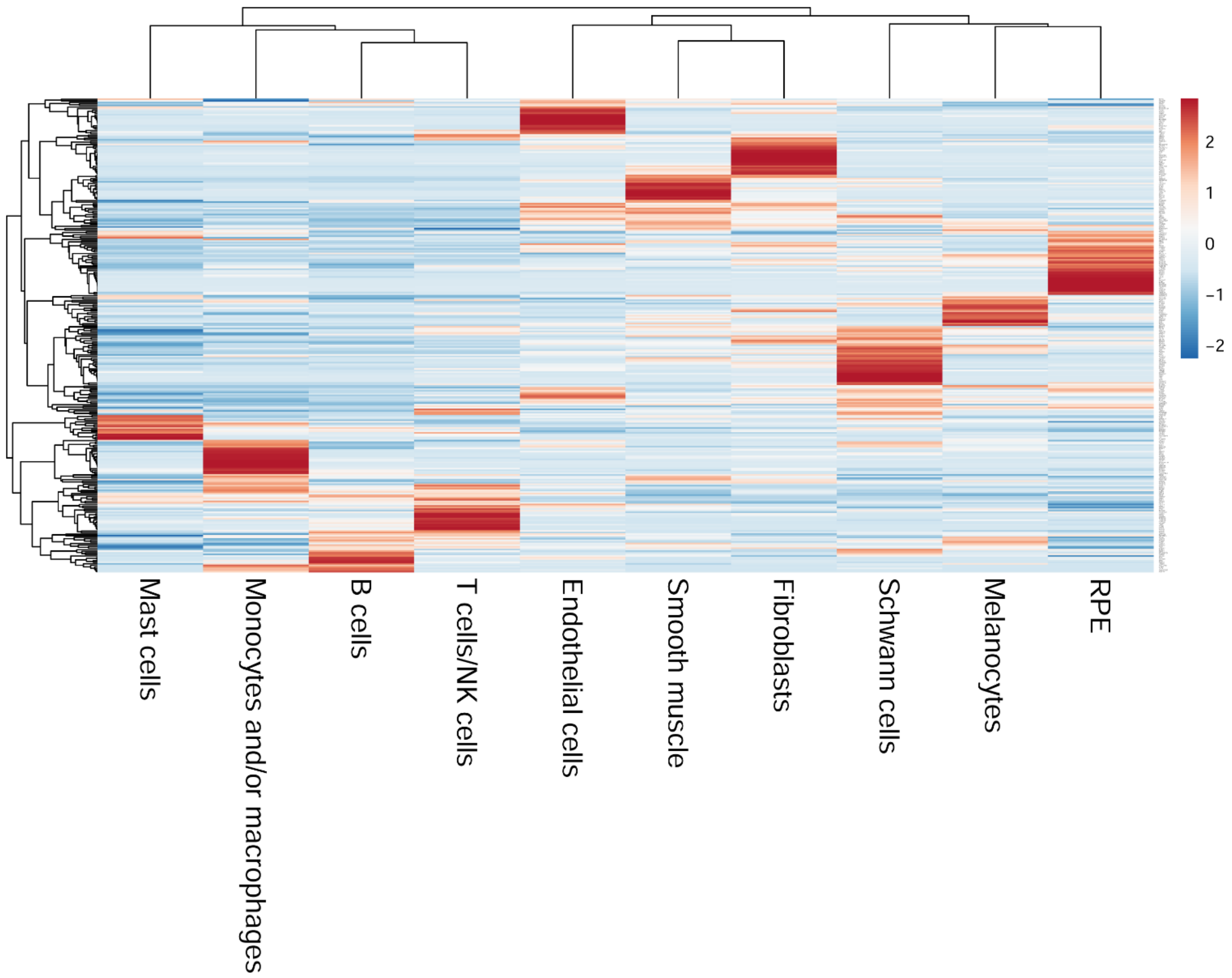

Supplementary Fig. S5. Clustering analyses of gene expression in 10 choroid/RPE cell types (using the Voigt et al, 2019 dataset). The map was generated using the ClustVis webtool (https://biit.cs.ut.ee/clustvis/).
