# Supplementary material for: Combining Gene–Disease Associations with Single-Cell Gene Expression Data Provides Anatomy-Specific Subnetworks in Age-Related Macular Degeneration
Source: Netw Syst Med. 2020 Aug 3;3(1):105–21. doi: 10.1089/nsm.2020.0005 (PMC7416628; doi:10.1089/nsm.2020.0005)
Supplement: Supplemental data [file Supp_Fig7.pdf]

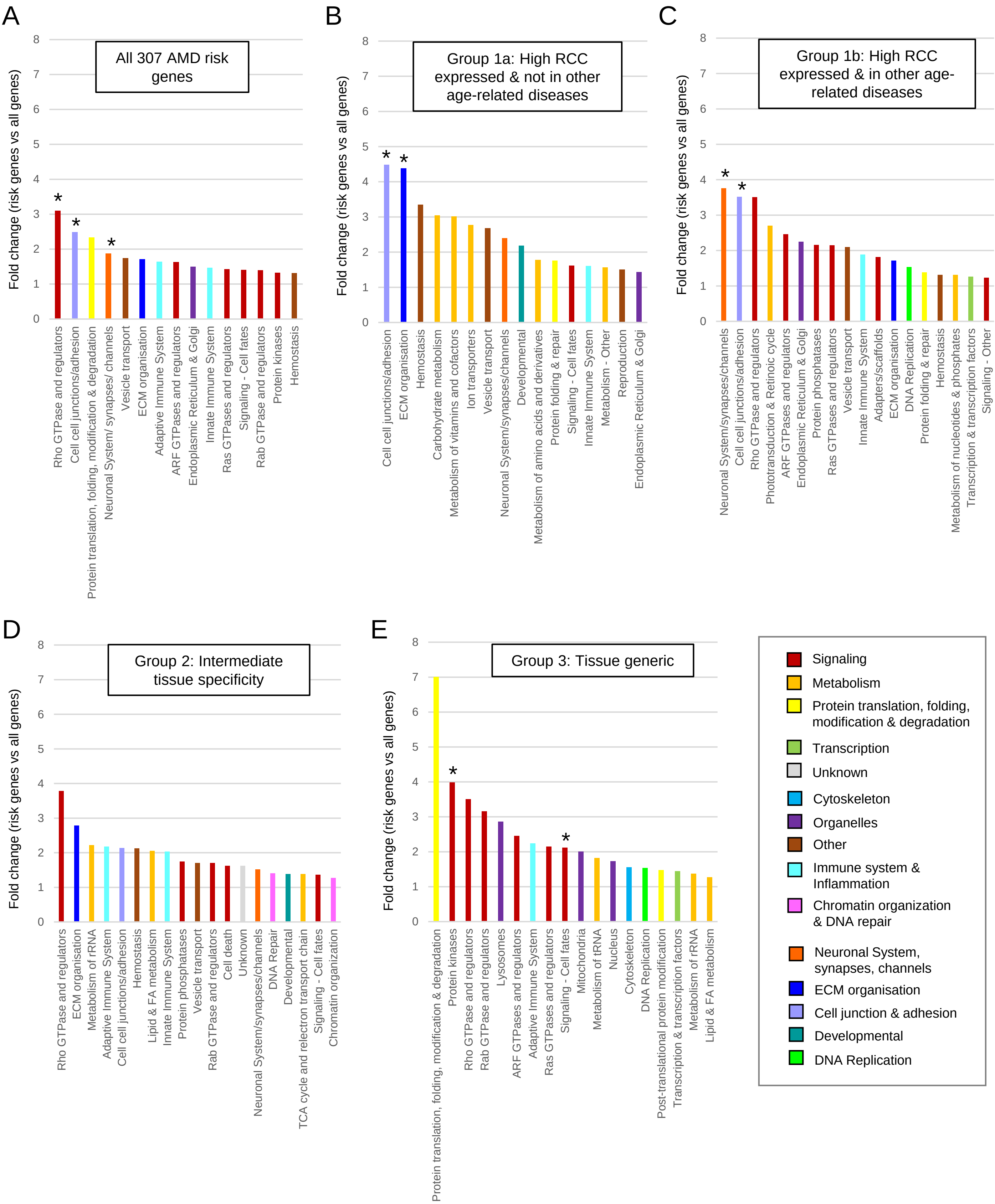

Supplementary Fig. S7. SysGO classes and enrichments for all 307 AMD risk genes (**A**) and sub group 1a (**B**), group 1b (**C**), group 2 (**D**), and group 3 (**E**) (see Supplementary Table S5). SysGO - set 2 is displayed. A star indicates significant p-values (<0.05) by Fisher's exact test.
