# Supplementary material for: Combining Gene–Disease Associations with Single-Cell Gene Expression Data Provides Anatomy-Specific Subnetworks in Age-Related Macular Degeneration
Source: Netw Syst Med. 2020 Aug 3;3(1):105–21. doi: 10.1089/nsm.2020.0005 (PMC7416628; doi:10.1089/nsm.2020.0005)
Supplement: Supplemental data [file Supp_Fig8.pdf]

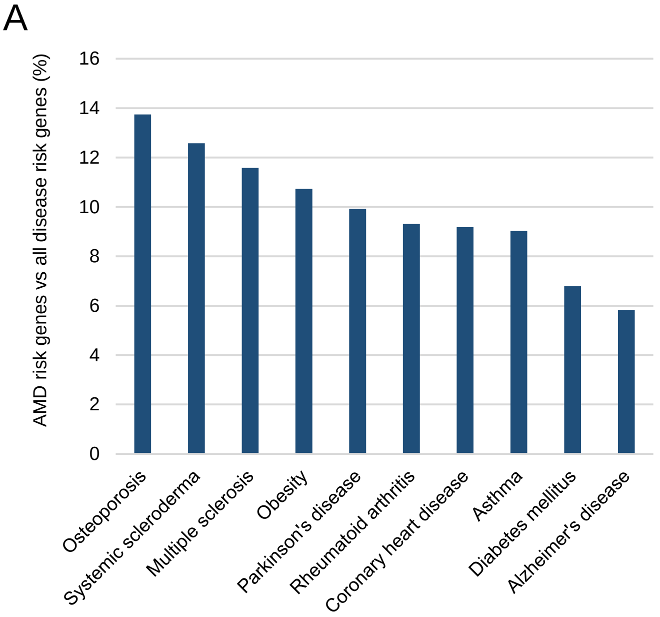

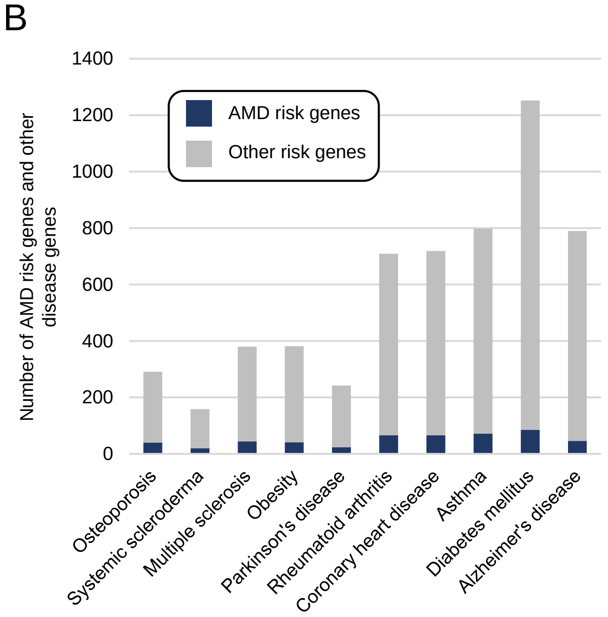

C

**Gene Symbol**	**Process (2)**	**Coronary heart disease**	**Diabetes mellitus**	**Rheumatoid arthritis**	**Alzheimer's disease**	**Obesity**	**Multiple sclerosis**	**Asthma**	**Systemic scleroderma**	**Osteoporosis**	**Parkinson's disease**
HLA-DQB1	Adaptive Immune System	X	X	X			X	X	X	X	X
IL2RA	GPCR signaling	X	X	X		X	X	X	X	X	
ETS1	Transcription & transcription factors		X	X	X		X	X	X	X	X
HLA-DRA	Adaptive Immune System		X	X	X		X	X	X	X	X
ADAMTS9	ECM organisation	X	X		X	X		X		X	X
CDH13	Cell_cell_junctions_adhesion	X			X	X	X	X	X		
ASAP2	ARF GTPases and regulators			X	X		X	X		X	
CETP	Lipid & FA metabolism	X	X			X		X			X
KCNQ1	Neuronal System_synapses_channels		X			X	X	X	X		
IKZF1	Transcription & transcription factors	X	X	X			X			X	
TBX3	Transcription & transcription factors	X	X		X	X				X	
TFAP2B	Transcription & transcription factors	X	X		X	X				X	
HLA-B	Adaptive Immune System		X	X			X	X	X		
LPP	Cell_cell_junctions_adhesion		X	X		X	X	X			
ABCA1	Lipid & FA metabolism	X	X		X	X				X	
PLEKHA1	Lipid & FA metabolism	X	X	X			X	X			
TYK2	Protein kinases		X	X			X		X	X	
JAZF1	Transcription & transcription factors		X	X			X	X	X		

Supplementary Fig. S8. Overlap of AMD risk genes with other age-related diseases. (**A**) Percentage of AMD risk genes in all risk genes per age-related disease. (**B**) Number of AMD risk genes and other age-related disease risk genes. (**C**) Genes with highest number (5 and higher) of associations with other age-related diseases.
