# Supplementary material for: Combining Gene–Disease Associations with Single-Cell Gene Expression Data Provides Anatomy-Specific Subnetworks in Age-Related Macular Degeneration
Source: Netw Syst Med. 2020 Aug 3;3(1):105–21. doi: 10.1089/nsm.2020.0005 (PMC7416628; doi:10.1089/nsm.2020.0005)
Supplement: Supplemental data [file Supp_Fig9.pdf]

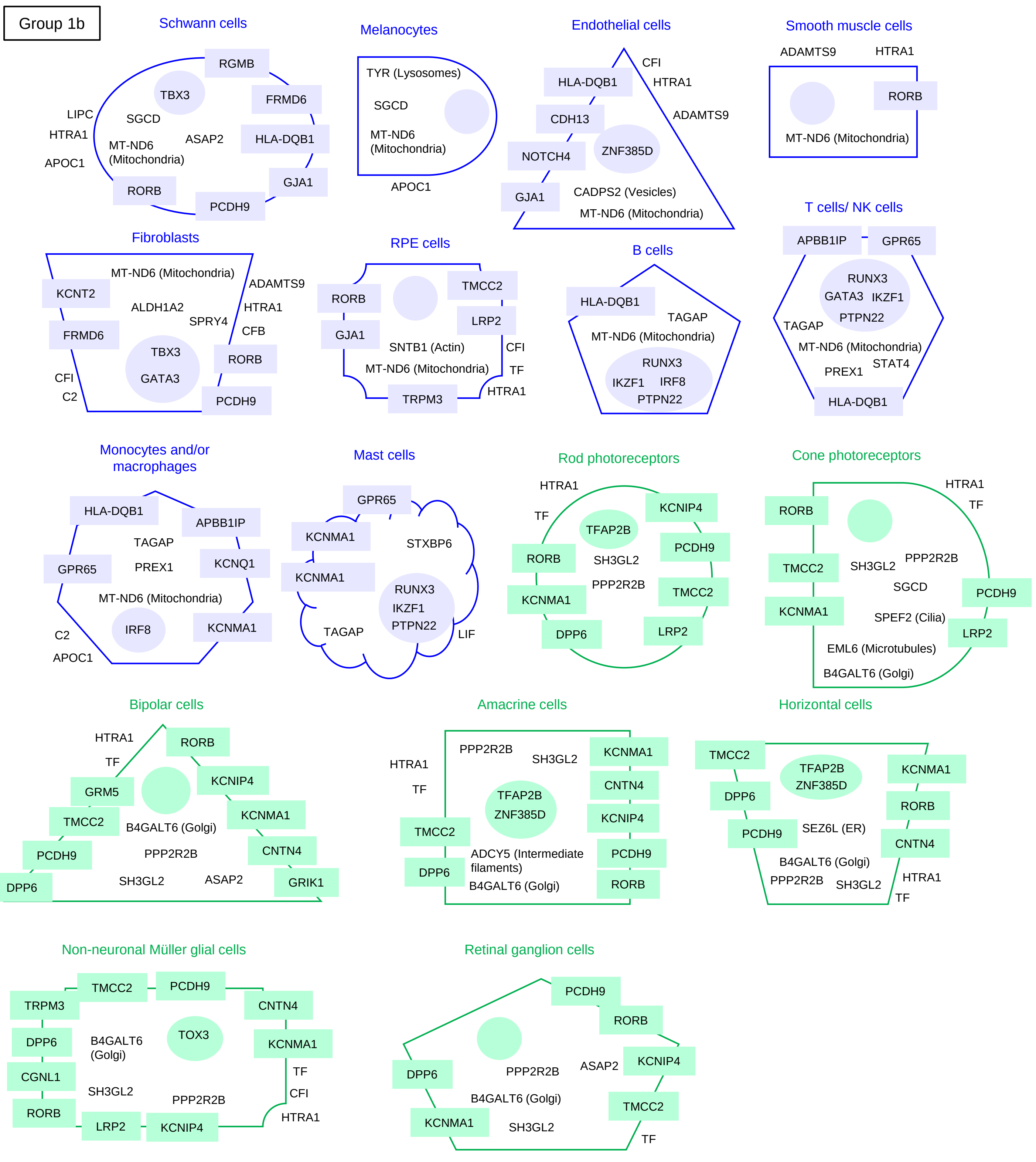

Supplementary Fig. S9. Subcellular localisation of group 1b genes that are highly expressed (expression groups A and B) in 17 cell types. Each cell types is represented using a distinct geometrical shape and colour (blue = choroid/RPE cells, and green = NR cell types) (see Fig. 2). Filled squared indicate membrane association, gene names outside the cell indicate extracellular localisation, inside the cell indicate cytosolic localisation, and within the filled circle indicate nuclear localisation. Other cellular/organelle localisations are indicated.
