# Supplementary material for: Combining Gene–Disease Associations with Single-Cell Gene Expression Data Provides Anatomy-Specific Subnetworks in Age-Related Macular Degeneration
Source: Netw Syst Med. 2020 Aug 3;3(1):105–21. doi: 10.1089/nsm.2020.0005 (PMC7416628; doi:10.1089/nsm.2020.0005)
Supplement: Supplemental data [file Supp_Fig11.pdf]

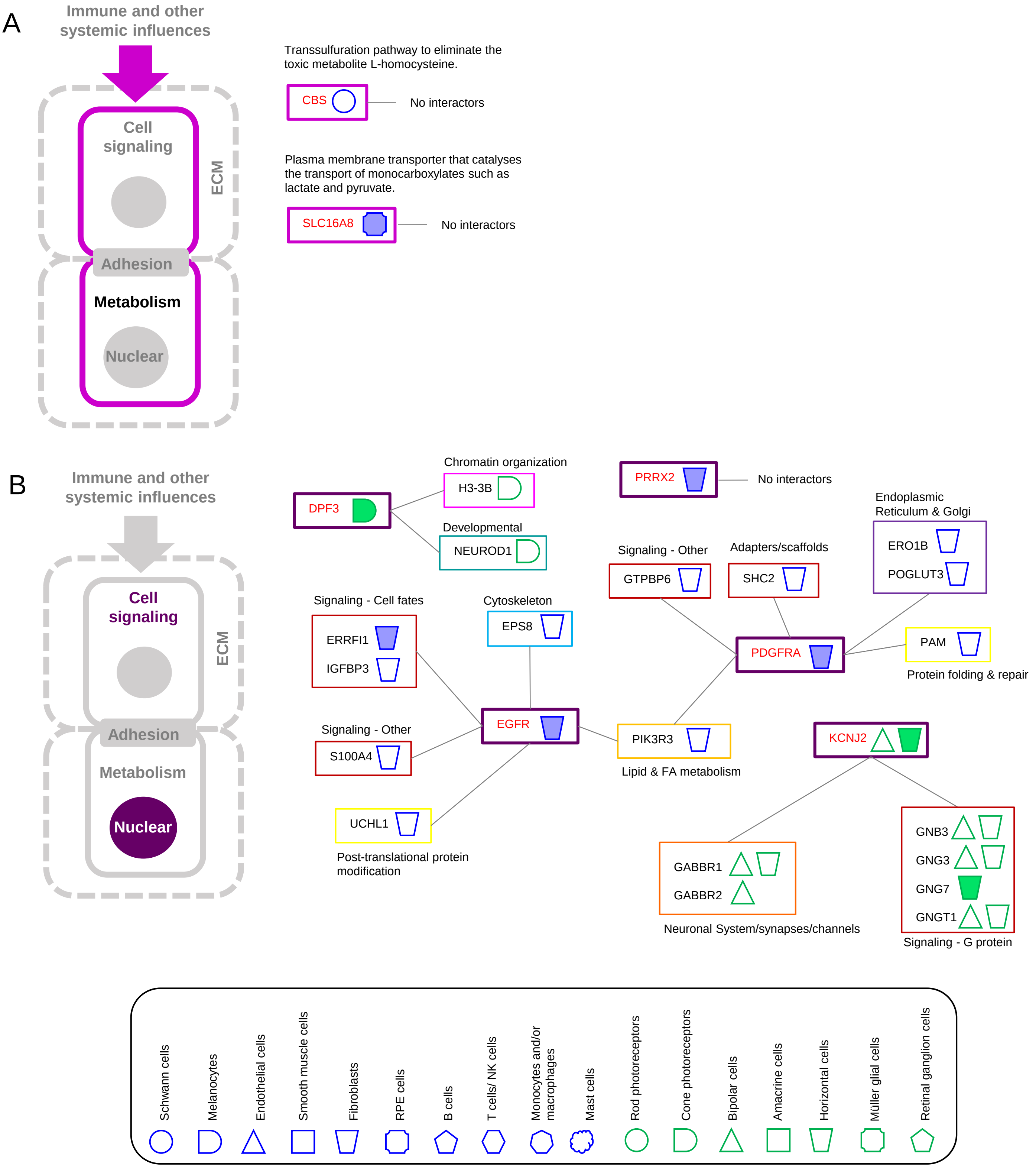

Supplementary Fig. S11. PPI networks of AMD risk protein of the anatomical layers AL5 (**A**) and AL6 (**B**). AMD risk proteins are indicated in red and the colour around the name corresponds to the AL. Interactors are grouped based on SysGO classes and linked to the AMD risk protein. The cell types in which the AND risk genes are highly expressed (expression groups A and B) are indicated using the geometrical shapes used before and as indicated in the legend. Filled geometrical shapes indicate z-scores of ≥ 2 for a specific gene when compared to its gene expression in the other cell types of the respective dataset (Voigt or Liang).
